# The *p*-wave superconductivity in the presence of Rashba interaction in 2DEG

**DOI:** 10.1038/srep29919

**Published:** 2016-07-26

**Authors:** Ke-Chuan Weng, C. D. Hu

**Affiliations:** 1 Department of Physics, National Taiwan University, Taipei 10617, Taiwan; 2 Center for Quantum Science and Engineering, National Taiwan University, Taipei 10617, Taiwan; 3 Research Center for Applied Sciences, Academia Sinica, Taipei 11529, Taiwan

## Abstract

We investigate the effect of the Rashba interaction on two dimensional superconductivity. The presence of the Rashba interaction lifts the spin degeneracy and gives rise to the spectrum of two bands. There are intraband and interband pairs scattering which result in the coupled gap equations. We find that there are isotropic and anisotropic components in the gap function. The latter has the form of cos *φ*_**k**_ where 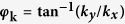. The former is suppressed because the intraband and the interband scatterings nearly cancel each other. Hence, −the system should exhibit the *p*-wave superconductivity. We perform a detailed study of electron-phonon interaction for 2DEG and find that, if only normal processes are considered, the effective coupling strength constant of this new superconductivity is about one-half of the *s*-wave case in the ordinary 2DEG because of the angular average of the additional 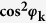 in the anisotropic gap function. By taking into account of Umklapp processes, we find they are the major contribution in the electron-phonon coupling in superconductivity and enhance the transition temperature *T*_*c*_.

Spin-orbit interaction (SOI) plays crucial roles both in opening a new field such as topological insulators^1^,^2^,^3^ and important applications on spin transport electronics named spintronics^4^,^5^,^6^. Of particular interest is the Rashba interaction^7^,^8^. It has often been studied in two-dimensional electron gas (2DEG) with a normal electric field created by the interface. For the semiconductor heterostructure, the effective electric field is created by confining potential. Gate voltage can be used to control the effective electric field^9^,^10^. Thus, the strength of Rashba interaction in the heterostructures can be tuned with the gate voltage. This provides a possibility to manipulate electron spins by electrical means.

Comparatively, Rashba interaction strength is usually weaker in semiconductors and stronger in the surface states of high Z metals, such as Au^11^, Bi^12^ and Pb^13^ due to stronger SOI induced orbital splitting. For a high Z metal film grown on the substrate, the inversion symmetry breaking in the direction perpendicular to 2DEG plane would induce the Rashba field^14^,^15^. The Rashba strength of quantum well state in the ultra-thin Pb film grown on Si(111) shows no strong dependence on coverage thickness^16^ and can be tuned through Si-doping^15^.

Recently, it was found that systems with Rashba interaction exhibit two-dimensional (2D) superconductivity, such as the interface of the LaAlO_3_/SrTiO^17^ and LaTiO_3_/SrTiO_3_^18^ heterostructures, and the interface of a topological insulator Bi_2_Te_3_ film grown on a non-superconducting FeTe thin film^19^. Furthermore, the superconductivity in Pb film from monolayer to ten layers grown on Si(111) were observed subsequently^20^,^21^,^22^. The oscillation of superconducting transition temperature *T*_*c*_ with the thickness of lead film were also reported^20^,^21^.

Theoretically, the effect of SOI using Green’s function approach was discussed in refs 23,24. Gor’kov and Rashba proposed that SOI would mix the spin-singlet and spin-triplet superconductivity^24^. The mixing of the spin-singlet d-wave and the spin-triplet p-wave due to inversion symmetry broken Rashba-type spin-orbit interaction via Hubbard model was reported in ref. 25. The *d*_*xy*_ + *p*-wave and 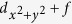-wave superconductivity in noncentrosymmetric systems were investigated and the new type of Andreev bound state was proposed in these systems^26^. In addition, the Andreev bound state and the Majorana edge mode that appeared in *d*_*xy*_ + *p*-wave case were also discussed in refs 26,27. The topological properties in nodal nocentrosymmetric superconductor were analyzed and the zero-energy flat band would give rise to certain topological features^28^. The enhancement of superconductivity due to spin-orbit interaction in the repulsive fermion gas was suggested by Vafek and Wang^29^. Topological superconductivity with the Majorana edge channels was suggested to appear in noncentrosymmetric superconductors^30^. For electron system with Rashba interaction, in addition to charge plasmon, the chiral spin modes and their mutual coupling were investigated in ref. 31. Electron transport in *p*-wave superconductor-normal metal junctions affected by interface SOI was studied in ref. 32. It was suggested that certain *p*-wave electron pairs can be tuned via the SOI and tunnel to the normal metal at a distance longer than mean free path of singlet pairing electrons.

In the presence of the Rashba interaction, the superconducting gap function depends on momentum ***p*** through its phase, Δ_p_ = exp(−*iφ*_p_)Δ_0_ where Δ_0_ is an isotropic gap energy, as derived in ref. 24. However, an approximation on the interaction potential had been made. We find that without the approximation, the magnitude of gap function is modulated by the extra cos *φ*_**p**_ factor and is anisotropic. We also make a detailed analysis of the effective interaction between electrons mediated by phonons. The results is summarized as the following. There are cancellations between different channels of scattering if the interaction is spin-independent. Approximation has to be made carefully in order to isolate the terms of cancellation. As a result, we find that the gap function is not only gauge-dependent but also has a factor 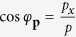. Thus, the *p*-wave gap dominates in the presence of Rashba interaction. The interaction strength of *p*-wave superconductivity is only half of that of conventional BCS *s*-wave superconductivity. Hence, its calculated *T*_*c*_ is as low as 0.6 K for lead film if only normal processes are considered. Only by including Umklapp processes, our results are comparable with experimental results.

This article is organized as following.The first section is the introduction. In the second section, we analyze Hamitonian in the Rashba eigen-spinor basis and obtain two coupled gap equations. In the third section, the gap equations are analyzed. The phonon mediated interaction and direct Coulomb interaction contributions are discussed separately. The corresponding dimensionless coupling strength constants *λ* and μ* are defined while solving the gap equations and they can be estimated by generalizing the discussion in ref. 33 to the 2DEG case. In the fourth section, we estimate the effective electron-phonon coupling constant by the model proposed by Scalapino *et al*. under the strong coupling approximation^34^,^35^,^36^ and Umklapp processes are considered. In the fifth section, we suggest that the *p*-wave superconductivity can be oberved in certain experiments. A conclusion is given in the last section.

## The Effect of Rashba Interaction in Superconductivity

In this section, we derive the effective Hamiltonian of superconductivity in the Rashba eigen-spinor basis. By diagonalizing the effective Hamiltonian, we can write down the ground state wave function and obtain the two coupled gap equations.

### Hamiltonian in the Rashba eigen-spinor basis

The 2D model Hamiltonian for the system with screened Coulomb interaction and electron-phonon interaction in the second quantization form is given by


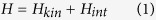


Here


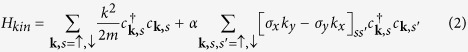


contains the kinetic energy and Rahsba interaction.


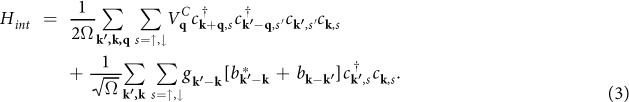


Ω is the area of the system. The first term of *H*_*int*_ is the Coulomb interaction between electrons.


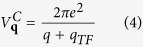


is the 2D screened electrostatic Coulomb potential energy where *q*_*TF*_ is the Thomas-Fermi wave vector and is given by


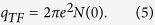


*N*(0) is the electron density of states at the the Fermi level. The second term of *H*_*int*_ is the electron-phonon coupling where 
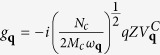
. *N*_*c*_ is the atomic density, *Z* is the valence of the ions, *M*_*c*_ is the mass of an ion and *ω*_***q***_ is 2D dressed phonon frequency.

The Rashba interaction mixes the spin-up and spin-down states of the free electrons. The eigenstates of *H*_*kin*_ are


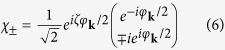


where 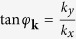 and *ζ* is a constant. The corresponding eigen-energies are


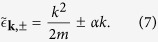


We note that the spinor basis functions can have various choices of phase and that used by Gor’kov and Rashba in ref. 24 had *ζ* = 1. Since the eigenstates are mixed states, we should write the Hamiltonian in terms of the Rashba eigen-spinors basis which will be referred as Rashba basis from now on.

The kinetic part *H*_*kin*_ can be diagonalized in the Rashba basis by using the following transformation


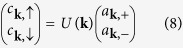


where


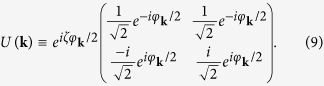



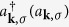
 is the creation(annihilation) operator of the electron in the *σ* band with momentum **k**. *σ* = + and *σ* = − represent the *χ*_+_ and *χ*_−_ spinor states respectively. These second quantized operators satisfy the commutation relation 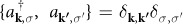, and 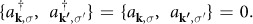 The kinetic energy of the system relative to the Fermi level *μ* is


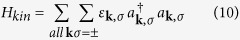


where 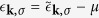. Combining the Coulomb interaction and electron-phonon interaction, the effective electron-electron interacting Hamiltonian is given as


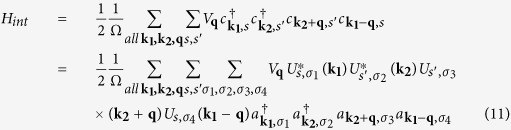


and can be written in the Rashba spinor basis. The explicit form of the interaction Hamiltonian in the Rashba spinor basis is shown in Appendix A of Supplementary Information. Here


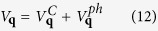


where 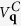 is the screened Coulomb potential in Eq. (4) and the phononmediated potential energy 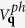 is


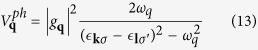


**Figure 1:**
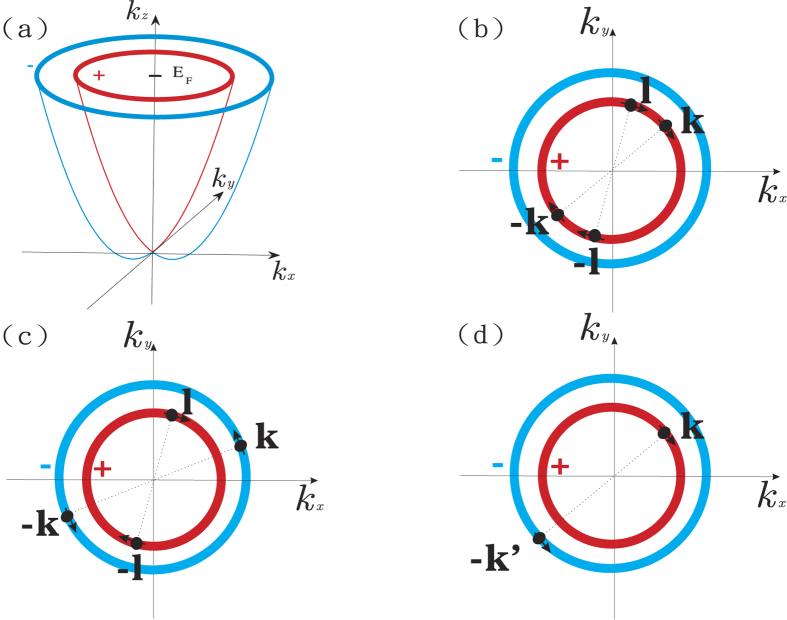
(**a**) The Rashba interaction lifts the spin degeneracy. As a result, the eigenstates are *σ* = +(blue color line) and *σ* = −(red color line) bands. (**b**–**d**) are the bands at the Fermi energy. In (**b**,**c**), the two electrons which form a pair can both be in *σ* = + band or in *σ* = −band. (**b**) An example of the intraband scattering. Both pairs involved in the scattering are in the *σ* = +band. (**c**) One pair involved in the scattering is in *σ* = +band and the other pair is in the *σ* = −band. This is the interband pairs scattering. (**d**) The pair formed by electrons from different bands is unstable because there is no other pairing state that such paired electrons can scattered into.

In the effective interaction Hamiltonian, there are interband and intraband interaction. However, while considering the scattering of the pairing electrons near the Fermi surface, the most important pairing configuration is that paired electrons being in the same band. The spinor bands and pairing of electrons are shown in Fig. 1. The zero-momentum pairing states uniformly distributed in either one of the two Rashba bands at Fermi level as shown in Fig. 1(b,c). The electrons in the same band can be scattered into any other unoccupied pairing states of zero momentum. This kind of scattering is the dominant scattering channel in superconductivity. Two electrons from different bands cannot form a pair with zero-momentum as shown in Fig. 1(d). There are much less states near the Fermi surface that such non-zero-momentum paired electrons can scattered into. Thus, such pairing composed of two electrons in different bands is of little importance. Hence we preserve only the terms with the pairing of electrons in the same band. The effective Hamiltonian involved in superconductivity is


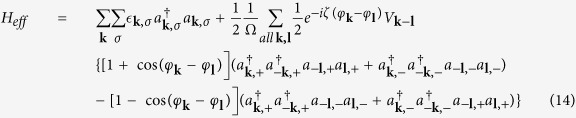


The second and third terms represent the intraband and interband pair scattering respectively. Here, the intraband scattering means interacting Cooper pairs stay in the same band and interband scattering means interacting Cooper pairs are in the different bands as shown in Fig. 1(b,c). We note that there are factors 1 ± cos(*φ*_*k*_ − *φ*_*l*_) in the second and third terms. These factors favor *p*-wave superconductivity as shown below.

### The two-band coupled gap equations

The Hamiltonian in Eq. (14) is to be diagonalized. The expectation value of pair creation operators on the ground state is defined as


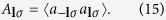


The Hamiltonian in Eq. (14) is expanded with respect to the fluctuations (*a*_−***l**σ*_*a*_***l**σ*_ − *A*_***l**σ*_) up to first order. Then we obtain


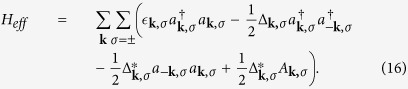


where


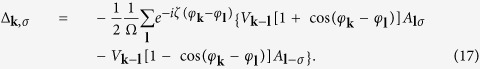


Δ_***k***,*σ*_ is an odd function of ***k*** because of *A*_−***l**σ*_ = −*A*_***l**σ*_. To diagonalize the Hamiltonian, we use the the Bogoliubov-Valentin transformation


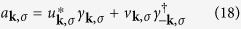


with constraints |*u*_***k***,*σ*_|^2^ + |*ν*_***k***,*σ*_|^2^ = 1, *u*_−***k***,*σ*_ = *u*_***k***,*σ*_ and *v*_−***k***,*σ*_ = −*v*_***k***,*σ*_^37^. The Fermi statistics of the *γ* operators satisfy 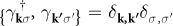, and 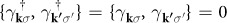. The mean field Hamiltonian in Eq. (16) can be diagonalized as


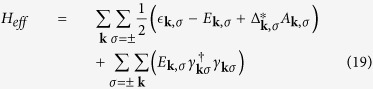


where 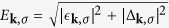 is the excitation energy of quasi-particles. Δ_***k***,*σ*_ is the gap energy. 
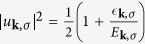
 and 
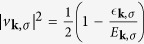
. Hence, the ground state wave function is


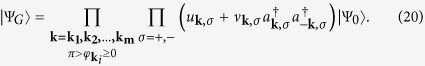


We note in passing that 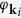 should be in the range 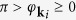 in ground state wave function, or the ground state can not be properly normalized. If the range of *φ*_*k*_ is from 0 to 2*π*, then the wave function


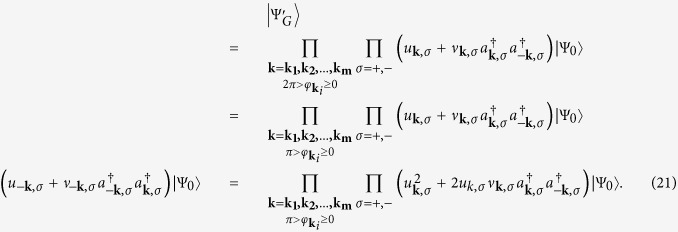


Clearly,


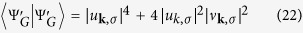


and 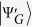 is not normalized.

The zero temperature gap equation can also be obtained by minimizing the expectation value of the effective Hamiltonian 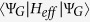, i.e., the derivative of 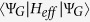 is equal to zero as the standard BCS process. There are two coupled gap equations


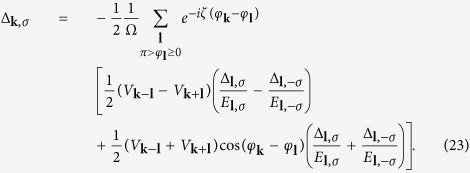


Because Δ_***k***,*σ*_ is an odd function of ***k***, it can also be written as


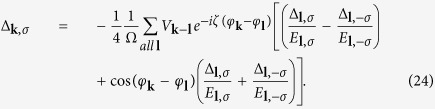


**Figure 2:**
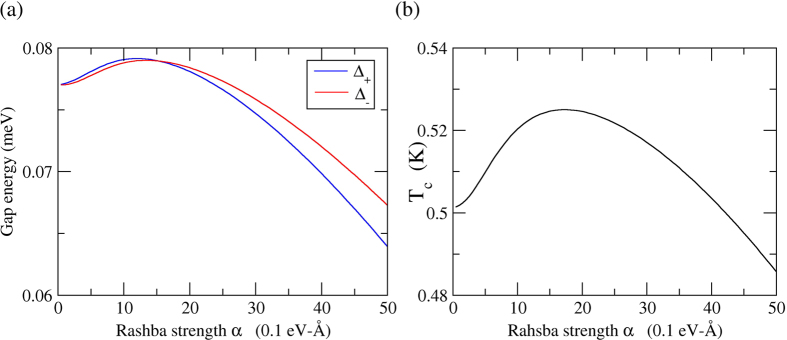
The Rashba effect in superconductivity for the Pb-film. The coupling strength is estimated by the model approximation in the absence Umklapp process. (**a**) The relation between the gap energy Δ_*σ*_ and Rashba strength *α* where the *p*-wave gap energy is Δ_*k*,*σ*_ = Δ_*σ*_cos *φ*_*k*_ and *σ* = ±. (**b**) The relation between the critical temperature *T*_*c*_ and Rashba strength *α*.

The detail derivation of Eq. (24) is in Appendix B of Supplementary Information. The 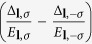 factor in the first summation term means the cancellation between intraband and interband pair scattering contribution and its contribution to the gap energy almost vanishes. It will be shown later in Fig. 2 that it is indeed this case. Hence the gap equation Eq. (24) can be approximated by


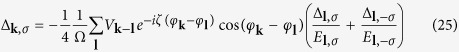


where 
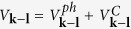
 as in Eq. (12).

### Comparison with previous theoretical investigation. 

In order to compare with the result in ref. 24, we derive the complete gap equations following the process of derivation in ref. 24. The Rashba eigen-spinor in Eq. (6) is used in the derivation. By taking *ζ* = 1 in Eq. (6), it can return to the spinor basis used in ref. 24.

The Hamitonian is


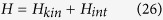


where


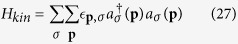


and


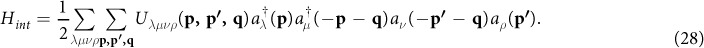


As shown in ref. 24.


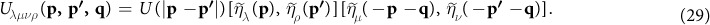


where *U*(|**p** − **p′**|) is the interaction strength and the scalar product of spinors is equal to


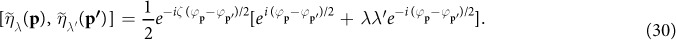


The same definition of Green functions in ref. 24 read


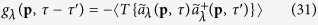



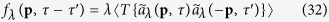



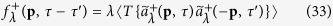


where 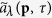 is *a*_*λ*_(***p***) in the Heisenberg representation. With *τ* − *τ*′ = 0^+^, we find


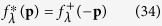


and


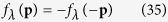


The same processes were given in Eqs (7, 8, 9, 10, 11, 12, 13) of ref. 24. *f*_*λ*_(**p**) can be related to the mean field *A*_**p***λ*_ we used in Eq. (15),


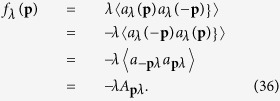


The Gor’kov equations are obtained as


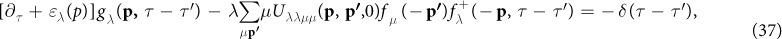



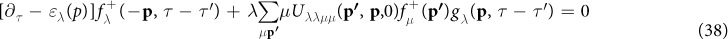


where


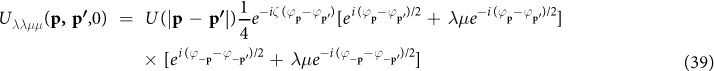


From the Gor’kov equations in Eqs (37,38), the gap function without approximation can be written as


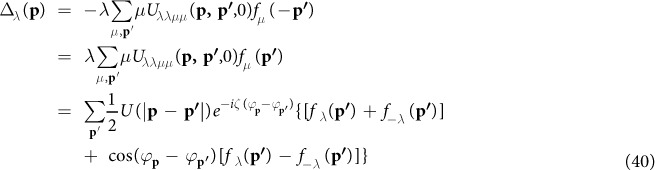


For *f*_*λ*_(**p**) = −*λA*_**p***λ*_, this gap function is identical with Eqs (17 and 24) in last section.

We return to the spinor basis in ref. 24 by taking *ζ* = 1. Eq. (40) becomes


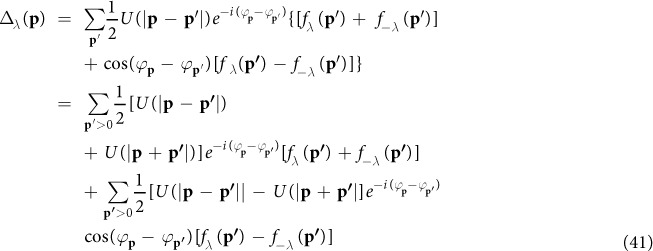


With the approximation *U*(|**p** − **p′**|) ≈ *U*(|**p** + **p′**|) ≈ *U*(0) in ref. 24, the term with the factor cos(*φ*_***k***_ − *φ*_***l***_) vanishes. We get


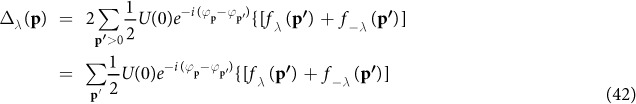


Thus we obtain the gap function Eq. (17) in ref. 24


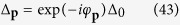



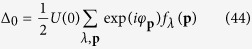


We have to note that both assumptions, *ζ* = 1 and *U*(|**p** − **p′**|) ≈ *U*(|**p** + **p′**|) ≈ *U*(0) are necessary for the cos(*φ*_***k***_ − *φ*_***l***_) term to vanish. But, the isotropic assumption *U*(|**p** − **p′**|) ≈ *U*(|**p** + **p′**|) ≈ *U*(0) is usually harmful to traditional triplet *p*-wave superconductivity and *p*-wave superfluidity^37^,^38^,^39^,^40^. The consequence of the approximation is the cancellation in Eq. (44). The definition of *f*_*λ*_(**p**, *τ* − *τ*′) in Eq. (32) results in a factor *λ* in front of the bracket. Hence, *f*_+_(**p**) and *f*_−_(**p**) have opposite signs. The summation over *λ* in Eq. (44) produces cancellation. The resulting gap function is greatly suppressed. Therefore, we concluded that the approximation *U*(|**p** − **p′**|) ≈ *U*(|**p** + **p′**|) ≈ *U*(0) of Eq. (14) in ref. 24 is not a good approximation for spin-independent interaction which we are dealing with in this work. On the other hand, the second term in the brace on the right hand side in Eq. (40) has two terms with opposite signs. We take advantage of that and reach Eq. (25). Our choice of phase of Rashba basis enable us to identify the possible cancellation as shown in Eq. (24). As a result, the gap equation Eq. (25) contains clearly the dominant interaction without any cancellation.

## The analysis of the interaction

In this section, we obtain the *p*-wave like gap energies (i.e., an additional cos(*φ*_**p**_) modulation to the gap function comparing with Eq. (43)) through the analysis of the gap equations. There are two dimensionless coupling constants. One is for electron-phonon interaction and the other is for electron-electron interaction. Following a procedure similar to the model proposed by Morel and Anderson^33^, one can evaluate the gap energies. The needed parameters can be found in Table 1. The finite temperature case and transition temperature are also discussed. The evolution of transition temperature relative to Rashba strength for Pb film on Si(111) is also shown.

### The superconducting state parameters

In this subsection, we give the outlines of how the electron-phonon interaction and Coulomb interaction are considered. The details can be found in Appendix C of Supplementary Information. For the free electron case, both spin-up and spin-down electrons have the equivalent contribution to the gap energy. However, in the presence of the Rashba interaction, the spin degeneracy is lifted and the gap energy is spin-band dependent.

From Eq. (25), the first term and second term in the brace of the summation are the intraband and the interband pair scattering contribution. We replace 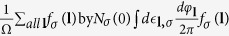. Here


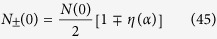


is the electron density of states for spin *χ*_±_ state at the Fermi energy and


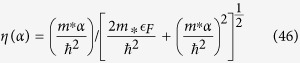


is the deviation of density of states due to Rashba splitting. Thus


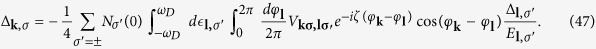


The gap energy


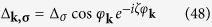


is used to solve gap equation Eq. (47).

We have to note that the more general form 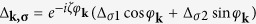 can usually be written as 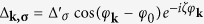 where 
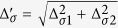
 and the cos(*φ*_***k***_ − *φ*_***l***_) in Eq. (47) can be written as cos(*φ*_***k***_ − *φ*_***l***_) = cos[(*φ*_***k***_ − *φ*_0_) − (*φ*_***l***_ − *φ*_0_)]. The solution will be similar to 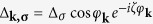 besides a phase shift *φ*_0_.

Small variation of Δ_***k***,***σ***_ in *E*_*k*,*σ*_ will be neglected such that 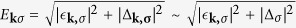 is used in Eq. (47). For 
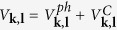
, we discuss the phonon mediated scattering process and the Coulomb interaction contribution to the gap energy separately. We use the model proposed by Morel and Anderson^33^ to analyze the parameters in the gap equations.

The effective potential of the phonon-mediated scattering, in view of Eq. (13) and Eqs (C3, C6, C7 and C10) in Appendix C of Supplementary Information. is


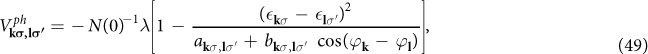


where


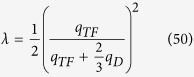


plays the role of dimensionless electron-phonon coupling constant “*N*(0)*V*” in BCS theory^41^,^42^. Here *q*_*D*_ is the Debye wave vector. Interested readers can find derivation in Appendix C of Supplementary Information. Here 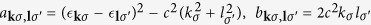 and *c* is the velocity of longitudinal phonon. Since *k*_*σ*_ and *l*_*σ*′_ are close to Fermi wave vector of *σ* and *σ*′ band respectively, |*a*_*kσ*, *lσ*′_| < |*b*_*kσ*, *lσ*′_| and the principle value 
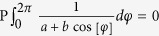
 for|*a*| < |*b*| will be applied.

The effective Coulomb interaction potential is taken as


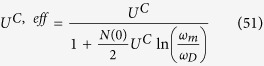


by Bogoliubov *et al*.^43^. Here 
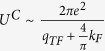
 is the average of the screened Coulomb potential *V*^*C*^ in 2D case as the discussion of Eq. (C14) in Appendix C of Supplementary Information. The dimensionless Coulomb coupling strength parameter


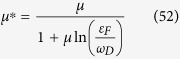


plays the role of the Coulomb psuedopotential in refs 33,43,44 where


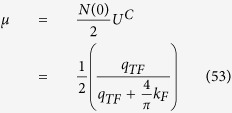


The gap equation, Eq. (47), including both electron-phonon and Coulomb interaction can be written as


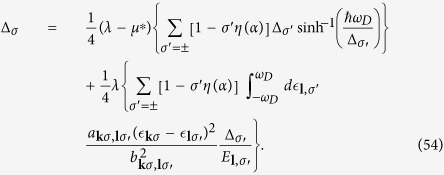


The dimensionless coupling strength in first term is composed of the sum of two bands and is roughly 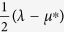 which is about one half of the ordinary 2DEG strength parameter (*λ* − μ*) as shown in Eq. (C17) in Appendix C of Supplementary Information. We have to note that the 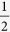 factor comes from the average of cos^2^ *φ*. The gap energy Δ_*σ*_ in the Rashba case should be smaller than in the free electron case due to the reduction of the coupling strength.

The first term in Eq. (54) dominates the right hand side of the equation. The last terms can be roughly estimated by taking 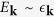 and 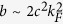. The integral for the last term is of the order 
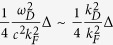
 which is much smaller than 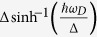 because 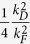 is usually much smaller than 1 and 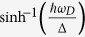 is usually larger than 1 in the numerical result.

### The finite temperature gap energy and transition temperature *T*_*c*_

The finite temperature gap energy equation can be obtained through Eqs (15) and (18). Substituting 
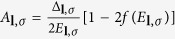
 where *f*(*E*_***l***,*σ*_) is the Fermi-Dirac distribution function in Eq. (17), we obtain


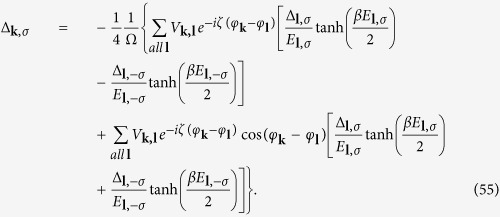


Similar to the zero temperature case, the interband and intraband pairs scattering contributions to the gap energy in the second summation dominate. The partial cancellation between interband and intraband pairs scattering in the first summation would reduce its contribution to the gap energy severely and can be neglected. Assuming a *p*-wave like gap energy 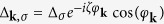 in Eq. (55) and replacing 
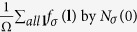
 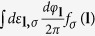, we obtain


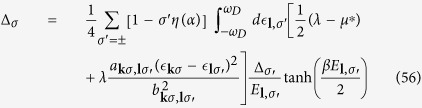


At transition temperature *T* = *T*_*c*_, Δ_*σ*_ → 0, it can be further simplified as


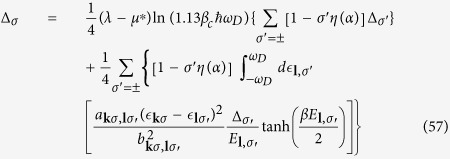


The first term usually dominates the right side of Eq. (57). Here again, the effective strength in the Rashba case is roughly half of the 2DEG case because of the angular dependence of Δ. As a result the critical temperature in the Rashba case becomes much smaller than that of conventional *s*-wave superconductor due to the exponential relation to the inverse of the coupling strength.

### Lead film

The Rashba effect^14^,^15^,^16^ and superconductivity^20^,^21^,^22^ had been reported seperately for the Pb film grown on Si(111) with *T*_*c*_ ranged from 1.5 K ~ 7K. Therefore, our model may be realized in lead thin film. The large effective mass of electrons in the quantum well state is taken to be 10 *m*_*e*_^45^. The lattice constatnt for Pb(111) film is 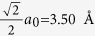
21 where *a*_0_ = 4.95 Å is the lattice constant of bulk lead. Thus the atomic density of the Pb(111) plane is 9.43 nm^−2 ^46, 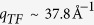
and 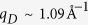 in the 2D case. All the parameters are list in Table 1. We solve Eq. (54) numerically for the case of Pb-film. The relation between gap energy and the Rashba strength *α* is shown in Fig. 2(a). In the Pb-film case, 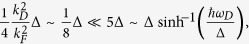 the first term in Eq. (54) surely dominates as we expect in section B. The relation between the transition temperature and Rashba strength for the Pb-film is also evaluated numerically and shown in Fig. 2(b). The estimated transition temperatures for bulk lead and lead film in which the normal processes are considered are shown in Table 1(b). We find that transition temperature *T*_*c*_ of *p*-wave superconductivity in the presence of the Rashba interaction is roughly 0.63 K as shown in Table 1(b). Hence, our calculation up to now can not account for the experimental finding in refs 20, 21, 22.

## 2-dimensional umklapp processes

In order to make perform a more accurate calculation and be able to explain exprimental results, we consider Umklapp processes in this section.

In the estimations of transition temperature and gap energy, the dimensionless electron-phonon coupling strength *λ* is an important factor. In 3-dimension case, Morel and Anderson proposed that *λ* is a state parameter and can be expressed as 
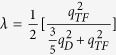
. In that approximation, *λ* is usually smaller than 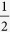 and is suitable for the weak coupling case. However, the Umklapp scattering was not included in that approximation and *λ* is usually underestimated^47^. For the strong-coupled superconductor, the self energy calculations is usually treated with the Eliashberg equation^48^,^49^ and the effective coupling strength can be properly renormalized. The transition temperature equation obtained by Macmillan^50^ can be applied to calculate of a number of metals and alloys. The electron-phonon coupling constant *λ* is defined as


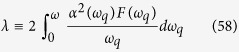


in Macmillan’s analysis^50^. Scalapino, Wada and Swihart^34^ estimated *α*^2^(*ω*_*q*_) by including both normal processes and umklapp contribution with appropriate *F*(*ω*_*q*_), and then evaluated the coupling strength *λ*. They were able to get results close to the experimental data^34^,^35^,^36^. In the previous section, we discuss the superconducting state parameter mainly following the model proposed by Morel and Anderson^33^ in the absence of the Umklapp processes. In this section, we estimate the 2D superconducting state parameters *λ* by revising the the model of Scalapino, Wada and Swihart^34^ for 2DEG.

The phonon coupling kernel


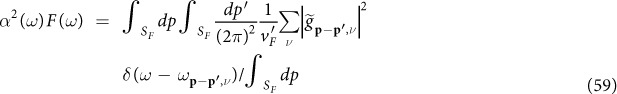


is used to evaluate *λ* in Eq. (58) for the 2D case. *S*_*F*_ is the Fermi surface and we assume **p** − **p′** = **q** + **K** where **K** is a reciprocal lattice vector. The electron-phonon matrix element is


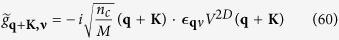


where *V*^2D^(**q** + **K**) is the effective Coulomb pseudo-potential and the phonon polarization is denoted by *ν*. The phonon density of states *F*(*ω*) is


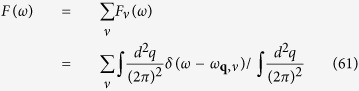


and the effective phonon coupling *α*^2^(*ω*) can be defined from Eqs (59) and (61). There is one longitudinal (*l*) mode and one transverse (*t*) mode for the phonon polarization and


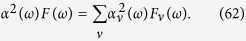


The first Brillouin zone is approximated by a circle of radius *q*_*D*_ and


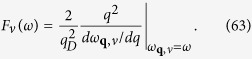


Thus the effective phonon coupling is


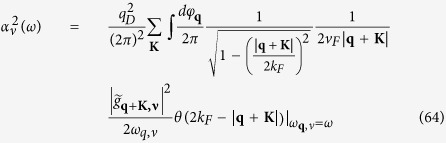


where **K** is a reciprocal lattice vector. Because electron-phonon coupling has contribution only when |**q** + **K**| < 2*k*_*F*_, there are 18 reciprocal lattice vectors **K** involved in the Umklapp processes listed in Table 2.

We follow the procedures in refs 34, 35, 36 to evaluate 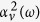 and *F*_*ν*_(*ω*) for lead. The potential in Eq. (60) of Harrison’s form is


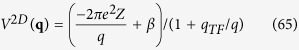


where *β* = 60 Ry-atomc unit of area 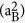 34. *F*_*ν*_(*ω*) is assumed to vary as *ω* at low frequency regime in order to obtain the linear dispersion. There are two peaks in phonon density of states, one transverse peak at 4.4 meV and one longitudinal peak at 8.5 meV for the bulk Pb. These peaks are also adopted in Pb-film case. We use the cutoff Lorentzians to approximate the peak of *F*_*ν*_(*ω*).


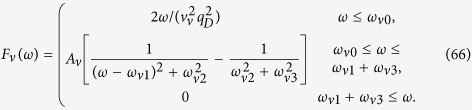


Normalization of *F*_*ν*_(*ω*) and continuity of *F*_*ν*_(*ω*) are used to determine *A*_*ν*_ and *ω*_*ν*0_. *ω*_*ν*3_ = 3*ω*_*ν*2_ is used in the Lorentzians. The parameters used in the calculation, the calculated coupling strength and the transition temperature for 2DEG are listed in Table 3. *α*^2^(*ω*) is a smooth function of *ω* and the value of *α*^2^(*ω*_*ν*0_) can be adopted for *α*^2^(*ω*) to evaluate *λ*. The effective phonon coupling strength *λ* = 1.05 and effective Coulomb interaction strength μ* = 0.1 for Pb film in 2DEG case. In this strong coupling case, the effective coupling strength is renormalized by *Z*(0) = 1 + *λ* and the renormalized coupling strength constants are 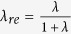 and 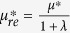.

**Figure 3:**
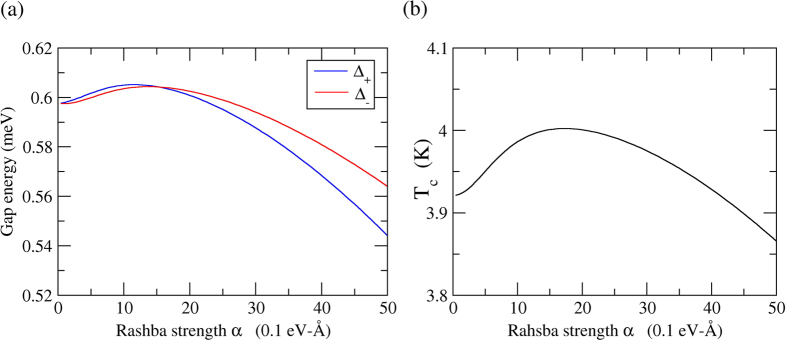
The Rashba effect in superconductivity for the Pb-film. The coupling strength is estimated by Scalapino, *et al*.’s model approximation. (**a**) The relation between the gap energy Δ_*σ*_ and Rashba strength *α* where the *p*-wave gap energy is Δ_*k*,*σ*_ = Δ_*σ*_cos *φ*_*k*_ and *σ* = ±. (**b**) The relation between the critical temperature *T*_*c*_ and Rashba strength *α*.

In the precence of Rashba interaction in 2DEG, the coupling strength constants are about one-half of those for the free electron case and *λ* = 0.525 and μ* = 0.05 are taken for the presence of Rashba interaction case. Considering the renormalization effect, the renormalized coupling strength constants *λ*_*re*_ = 0.344 and *μ*_*re*_ = 0.033 are adopted in Eqs (54) and (57), the relation between gap energy Δ and to Rashba strength *α* and transition temperature *T*_*c*_ relative to Rashba strength *α* are shown in Fig. 3(a,b) respectively. 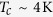 in the presence of Rashba interaction case. The experimental *T*_*c*_ ranged from 1.5~7 K for lead film on Si(111) are reported in refs 20, 21, 22.

We have to note that as shown in the Table 3, the transverse phonon coupling 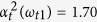 is larger than that of the longitudinal phonon coupling 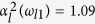. However, such transverse phonon mode is not included in the model estimation of last section in the absence of Umklapp process. The transverse mode which comes from the Umklapp processes has important contribution to the electron phonon coupling. Hence, the gap energy and transition temperature is enhanced while including the umklapp processes and the final result agrees reasonably well with experiments.

Finally, we discuss the effect of the band structure in lead. The strong-coupling superconductivity of lead was described very well by Eliashberg formalism^48^,^51^ with practical physical quantities such as phonon spectra and electron density of states. In that and subsequent treatments, the Fermi surface was assumed to be spherical like what we have done in this work. Later, lead was reported to be a two-band superconductor^52^,^53^, albeit with two very close energy gaps. Our calculation is a reasonable approximation in the case of lead. The argument is presented in Appendix E of of Supplementary Information.

## The observation of the *p*-wave superconductivity

We discuss how our calculation can be verified by experiments in this section. For the conventional superconductor, the excitation probability of the quasiparticles of isotropic gap energy Δ is proportional to exp[−Δ/*k*_*B*_*T*]. Thus, the power law T dependence of specific heat^54^ at 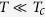 is inconsistent with isotropic gap prediction. This power law T dependence is due to the allowed states around the nodes in the superconducting gaps and is a feature of the unconventional superconductor. This can be applied to verify the nodes of this *p*-wave like gap energy.

Transverse ultrasound attenuation can be used in gap-anisotropic systems and probe the electronic gap nodes. By analyzing the quasiparticle contribution in the transverse ultrasound attenuation, the relationship between the quasiparticle gap structure and the electron viscosity tensor can be examined^55^. For temperature low enough, the quasiparticles are entirely concentrated within the gap nodes of the excitation spectrum. The attenuation due to certain node is related to the propagation direction and polarization direction of the sound wave. If neither direction is perpendicular to the node position vector in *k*-space, the attenuation is activated^55^. It had been used to locate the gap lines and nodes of anisotropic superconductor UPt_3_ 56. The ultrasonic attenuation measurements on *p*-wave superconductor Sr_2_RuO_4_ 57 and *d*-wave cuprate superconductors YBa_2_CuO_3+x _58 were also performed.

The tunneling spectroscopy were used to analysis gap profile in superconductivity. When an electron tunnels from a normal metal to an anisotropic superconductor, the tunneling depends on the angle between the superconducting crystal orientation and the interface because of the anisotropic gap^59^. For example, the zero-bias conductance peaks (ZBCPs) of a superconductor/insulator/normal metal tunneling conductance curve was reported in *d*-wave superconductors^60^,^61^ which is the consequence of the Andreev bound states^62^,^63^ and only exist in the interface of junction. There should also be clear ZBCPs in the crystal node orientations in our *p*-wave like superconductor junctions. It is different from the flat U-shape conductance curve with no ZBCP for the *s*-wave case.

In addition, for both the diffusive normal metal/*p*-wave superconductor junction and diffusive normal metal/*d*-wave superconductor juction, while the gap node direction is along the interface, the injected and reflected quasiparticles feel different sign of the pair potentials and mid-gap Andreev resonant state (MARS) form. The *p*-wave or *d*-wave superconductivity at such normal metal/unconventional superconductor junction still can be distiguished by the charge transport property^64^. In *d*-wave superconductor, the destructive angular average of the proximity effect in MARS would result in zero proximity. However, in *p*-wave superconductor, the destructive average of the proximity effect in MARS is avoid and the proximity is finite. The proximity effect can be investigated from the local density of states (LDOS) in the normal metal side of the normal metal/superconductor junction. In *p*-wave superconductor case, there should be zero energy peak of LDOS in the normal metal side because of the penetration of the MARS from the superconductor side into the normal metal region. In *d*-wave superconductor case, there are zero proximity in MARS and LDOS in the normal metal side is a constant and still flat at zero energy. This difference is suggested to originate from the symmetry of the induced odd frequency pairing^65^,^66^. Thus, the *p*-wave and *d*-wave superconductor can be distinguished.

## Conclusion

We investigate the effect in the superconductivity in the presence of the Rashba interaction. The presence of the Rashba field requires a new basis. Consider only the pairing in the same band, we obtain the coupled gap equations of two bands. Due to the partial cancellation between the intraband and interband pairs scattering, the dominant gap function is *p*-wave like 
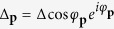
. In addition to the phase dependent gap function suggested in ref. 24, the magnitude of gap function is also modulated by cos(*φ*_**p**_) in our investigation. The factor cos*φ*_**p**_ gives rise to an extra factor of 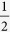 in the gap equation so that the dominant coupling strength parameter is 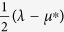 which is one half of that of the ordinary 2DEG case. As a result, the gap energy (transition temperature) would be an order of magnitude smaller than the 2DEG case if only the normal-process is considered.

The Pb film on Si(111) is a system where the Rashba interaction exist and can be the case analyzed in this article. We estimate the 2D coupling strength parameters by generalizing the 3D model estimation of Morel and Anderson^33^. Considering only the normal process of electron-phonon interaction, we find that *T*_*c*_ is of the order 0.6 K in the presence of the Rashba interaction. When the Umklapp processes are included, the coupling strength parameter may be larger than 1 and has to be renormalized in this strong coupling case. The calculated *T*_*c*_ is of the order 4 K in the presence of Rashba interaction. Our calculation shows that the Umklapp processes provide the major contribution to the electron-phonon interaction.

## Additional Information

**How to cite this article**: Weng, K.-C. and Hu, C. D. The *p*-wave superconductivity in the presence of Rashba interaction in 2DEG. *Sci. Rep.* **6**, 29919; doi: 10.1038/srep29919 (2016).

## Supplementary Material

Supplementary Information

## Figures and Tables

**Table 1:** 

		3D theoretical expression	Lead [bulk]	2D theoretical expression	Lead film [Pb/Si(111)]
**(a)**					
Electron-phonon coupling strength	*λ*	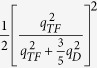 ^‡^	0.40^‡^	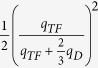	0.48
Angular averaged Coulomb coupling	*μ*	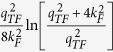 ^‡^	0.32	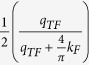	0.32
Effective Coulomb coupling strength (Coulomb psudopotential^‡^)	μ*	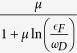 ^‡^	0.1^‡^	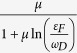	0.1
Lattice constant	*a*_0_		4.95 Å		3.50 Å⋇
Atomic density	*n*_*c*_	*N*_*c*_/*V*	33.0 nm^−3^	*Nc*/*A*	9.43 nm^−2*^
Fermi wave number	*k*_*F*_	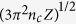	1.57 Å^−1†^	(2*πn*_*c*_*Z*)^1/2^	1.54 Å^−1^
Debye wave number	*q*_*D*_	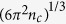	1.25 Å^−1†^	(4*πn*_*c*_)^1/2^	1.09 Å^−1^
Thomas Fermi wave number	*q*_*TF*_	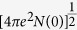 ^‡^	2.82 Å^−1^	2*πe*^2^*N*(0)	37.8 Å^−1^
Effective mass of electron	m*		2.1 *m*_*e*_^‡^		10 *m*_*e*_
Density of states (Fermi surface)	*N*(0)	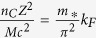	44.0 nm^−3^ eV^−1^	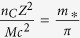	41.8 nm^−2^ eV^−1^
Velovity of longitudinal phonon [for phonon dispersion (*ω*_*q*_ = *cq*)]	*c*	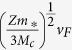	2.36 × 103 m/s	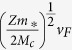	1.30 × 10^3^ m/s
Debye temperature	*ωD*		105 K		105 K

		**Transition temperature (*****Tc***)			
		**Theoretical value**	**Experimental value**		
**(b)**					
Lead bulk	Free electron	4.25 K 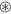	7.19 K		
	2DEG (without Rashba interaction)	8.62 K 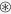			
Lead film			1.5 K~7 K		
	2DEG (with Rashba interaction)	0.63 K^⊙^			

(a) The 3D and 2D superconducting state parameters. The data with ‡, †, ⋇ and * indices are taken from refs 21,33,36,46 respectively. (b) The estimated transition temperatures for lead bulk and lead film in which the normal processes are considered. Without Rashba interaction, BCS transition temperature equation 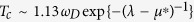 is used to estimate *T*_*c*_ for lead bulk and lead film and noted by 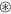. 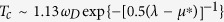 is used to estimate *T*_*c*_ in the presence of the Rashba interaction in 2DEG. It is approximated by preserving the first term in the right of Eq. (57) under the Δ_*σ*_ → 0 limit and the evaluated *T*_*c*_ is noted by ⊙. The experimental *T*_*c*_ for lead bulk is from ref. 67. The experimetal values for Pb film on Si(111) ranged from 1.5~7 K were reported in refs 20, 21, 22.

**Table 2:** Reciprocal lattice vectors K involve in the umklapp processes.

*K*(Å^−1^)	Number of *K*
1.79	6
3.11	6
3.59	6

**Table 3:** The parameters for phonon density of states used in Eq. (66) and electron-phonon coupling in Eq. (64).

		*ν*	*ω*_*ν*0_	*ω*_*ν*1_	*ω*_*ν*2_	*A*_*ν*_	*v*_*ν*_	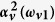	*λ*	μ*	*Z*(0)	*λ*_*re*_ = (*λ*)/(*Z*(0))	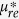 = (μ^*^)/(*Z*(0))	*Tc*
			(meV)	(meV)	(meV)	(meV)	(km/s)	(meV)						(K)
Lead bulk	Free electron	*t*	2.5^‡^	4.4^‡^	0.75^‡^	0.39^‡^	1.07^‡^	1.09	1.32	0.10	2.32	0.569	0.043	8.69
		*l*	7.1^‡^	8.5^‡^	0.50^‡^	0.25^‡^	2.42^‡^	1.26						
Lead film	2DEG (without Rashba interaction)	*t*	3.2	4.4	0.75	0.34	1.07	1.70	1.05	0.10	2.05	0.512	0.049	6.51
		*l*	7.3	8.5	0.50	0.22	2.42	1.09	0.525	0.05	1.525	0.344	0.033	~4
	2DEG (with Rashba interaction)													

The values with ‡ index are adopted from ref. 36. The adoption of phonon peak energy *ω*_*ν*1_, the width of Lorentzians *ω*_*ν*2_ and sound velocity *v*_*ν*_ for the lead film are the same as those of bulk lead. The Macmillan transition temperature equation 
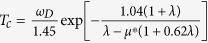
 50 is used to estimate *T*_*c*_ for the free electron case. The experimental *T*_*c*_ for bulk lead is 7.19 K which corresponds to *λ* = 1.12 and *μ* = 0.1. The Debye temperature *ω*_*D*_ is 105 K. Coupling strength constants *λ*, *μ* and the zero energy renormalization factor *Z*(0) = 1 + *λ* are also listed in Table. The renormalized coupling strength constants *λ*_*re*_ = 0.344 and *μ*_*re*_ = 0.033 are adopted in Eq. (57) for the presence of Rashba interaction case and the estimated *Tc*~4 K. The experimental *T*_*c*_ ranged from 1.5~7 K for lead film on Si(111) are reported in refs 20, 21, 22.
