# Supplementary material for: The p-wave superconductivity in the presence of Rashba interaction in 2DEG
Source: Sci Rep. 2016 Jul 26;6:29919. doi: 10.1038/srep29919 (PMC4961222; doi:10.1038/srep29919)
Supplement: Supplementary Information [file srep29919-s1.pdf]

The p-wave superconductivity in the presence of Rashba interaction in 2DEG

Ke-Chuan Weng[1,2,3] and and C. D. Hu[1,2,*]

[1]Department of Physics, National Taiwan University, Taipei 10617, Taiwan
[2]Center for Quantum Science and Engineering, National Taiwan University, Taipei 10617, Taiwan
[3]Research Center for Applied Sciences, Academia Sinica, Taipei 11529, Taiwan.

Supplementary Information

Appendix

Appendix A: The second quantized Hamiltonian in Rashba eigenbasis

The interaction Hamitonian, Eq. (11) can be written explicitly as

$$
\begin{aligned}
H_{int} =& \frac{1}{2}\frac{1}{\Omega}\sum_{\mathbf{k_1},\mathbf{k_2},\mathbf{q}}\sum_{\sigma=\pm} V_q e^{-i(\zeta/2)(\varphi_{\mathbf{k_1}}+\varphi_{\mathbf{k_2}}-\varphi_{\mathbf{k_1-q}}-\varphi_{\mathbf{k_2+q}})} \\
& [i\sin(\frac{\varphi_{\mathbf{k_1}}-\varphi_{\mathbf{k_1-q}}}{2})\cos(\frac{\varphi_{\mathbf{k_2}}-\varphi_{\mathbf{k_2+q}}}{2})(a^\dagger_{\mathbf{k_1},\sigma}a^\dagger_{\mathbf{k_2},\sigma}a_{\mathbf{k_2+q},\sigma}a_{\mathbf{k_1-q},-\sigma}+a^\dagger_{\mathbf{k_1},-\sigma}a^\dagger_{\mathbf{k_2},\sigma}a_{\mathbf{k_2+q},\sigma}a_{\mathbf{k_1-q},\sigma}) \\
& + i\cos(\frac{\varphi_{\mathbf{k_1}}-\varphi_{\mathbf{k_1-q}}}{2})\sin(\frac{\varphi_{\mathbf{k_2}}-\varphi_{\mathbf{k_2+q}}}{2})(a^\dagger_{\mathbf{k_1},\sigma}a^\dagger_{\mathbf{k_2},-\sigma}a_{\mathbf{k_2+q},\sigma}a_{\mathbf{k_1-q},\sigma}+a^\dagger_{\mathbf{k_1},\sigma}a^\dagger_{\mathbf{k_2},\sigma}a_{\mathbf{k_2+q},-\sigma}a_{\mathbf{k_1-q},\sigma}) \\
& + \cos(\frac{\varphi_{\mathbf{k_1}}-\varphi_{\mathbf{k_1-q}}}{2})\cos(\frac{\varphi_{\mathbf{k_2}}-\varphi_{\mathbf{k_2+q}}}{2})(a^\dagger_{\mathbf{k_1},\sigma}a^\dagger_{\mathbf{k_2},\sigma}a_{\mathbf{k_2+q},\sigma}a_{\mathbf{k_1-q},\sigma}+a^\dagger_{\mathbf{k_1},\sigma}a^\dagger_{\mathbf{k_2},-\sigma}a_{\mathbf{k_2+q},-\sigma}a_{\mathbf{k_1-q},\sigma}) \\
& - \sin(\frac{\varphi_{\mathbf{k_1}}-\varphi_{\mathbf{k_1-q}}}{2})\sin(\frac{\varphi_{\mathbf{k_2}}-\varphi_{\mathbf{k_2+q}}}{2})(a^\dagger_{\mathbf{k_1},\sigma}a^\dagger_{\mathbf{k_2},\sigma}a_{\mathbf{k_2+q},-\sigma}a_{\mathbf{k_1-q},-\sigma}+a^\dagger_{\mathbf{k_1},\sigma}a^\dagger_{\mathbf{k_2},-\sigma}a_{\mathbf{k_2+q},\sigma}a_{\mathbf{k_1-q},-\sigma})]
\end{aligned}
\tag{A1}
$$

Appendix B: Detailed derivation of Eq. (24)

In this appendix, we give a detailed derivation of Eq. (24) in the main text.
From Eq. (11)

$$
\begin{aligned}
H_{int} &= \frac{1}{2}\frac{1}{\Omega}\sum_{all\ \mathbf{k_1},\mathbf{k_2},\mathbf{q}}\sum_{s,s'}\sum_{\sigma_1,\sigma_2,\sigma_3,\sigma_4} V_\mathbf{q}\, U^*_{s,\sigma_1}(\mathbf{k_1})U^*_{s',\sigma_2}(\mathbf{k_2})U_{s',\sigma_3}(\mathbf{k_2+q})U_{s,\sigma_4}(\mathbf{k_1-q})a^\dagger_{\mathbf{k_1},\sigma_1}a^\dagger_{\mathbf{k_2},\sigma_2}a_{\mathbf{k_2+q},\sigma_3}a_{\mathbf{k_1-q},\sigma_4} \\
&= \frac{1}{2}\frac{1}{\Omega}\sum_{all\ \mathbf{k_1},\mathbf{k_2},\mathbf{q}}\sum_{\sigma_1,\sigma_2,\sigma_3,\sigma_4} V_\mathbf{q}\, [\sum_s U^*_{s,\sigma_1}(\mathbf{k_1})U_{s,\sigma_4}(\mathbf{k_1-q})][\sum_{s'} U^*_{s',\sigma_2}(\mathbf{k_2})U_{s',\sigma_3}(\mathbf{k_2+q})] \\
&\qquad a^\dagger_{\mathbf{k_1},\sigma_1}a^\dagger_{\mathbf{k_2},\sigma_2}a_{\mathbf{k_2+q},\sigma_3}a_{\mathbf{k_1-q},\sigma_4} \\
&= \frac{1}{2}\frac{1}{\Omega}\sum_{all\ \mathbf{k_1},\mathbf{k_2},\mathbf{q}}\sum_{\sigma_1,\sigma_2,\sigma_3,\sigma_4} V_\mathbf{q}\, [\sum_s U^\dagger_{\sigma_1,s}(\mathbf{k_1})U_{s,\sigma_4}(\mathbf{k_1-q})][\sum_{s'} U^\dagger_{\sigma_2,s'}(\mathbf{k_2})U_{s',\sigma_3}(\mathbf{k_2+q})] \\
&\qquad a^\dagger_{\mathbf{k_1},\sigma_1}a^\dagger_{\mathbf{k_2},\sigma_2}a_{\mathbf{k_2+q},\sigma_3}a_{\mathbf{k_1-q},\sigma_4} \\
&= \frac{1}{2}\frac{1}{\Omega}\sum_{all\ \mathbf{k_1},\mathbf{k_2},\mathbf{q}}\sum_{\sigma_1,\sigma_2,\sigma_3,\sigma_4} V_\mathbf{q}\, [U^\dagger(\mathbf{k_1})U(\mathbf{k_1-q})]_{\sigma_1\sigma_4}[U^\dagger(\mathbf{k_2})U(\mathbf{k_2+q})]_{\sigma_2\sigma_3}a^\dagger_{\mathbf{k_1},\sigma_1}a^\dagger_{\mathbf{k_2},\sigma_2}a_{\mathbf{k_2+q},\sigma_3}a_{\mathbf{k_1-q},\sigma_4}
\end{aligned}
\tag{B1}
$$

* cdhu@phys.ntu.edu.tw

Here

$$U^{\dagger}(\mathbf{k})U(\mathbf{l}) = e^{-i\zeta(\varphi_{\mathbf{k}}-\varphi_{\mathbf{l}})/2}\begin{pmatrix} \cos(\frac{\varphi_{\mathbf{k}}-\varphi_{\mathbf{l}}}{2}) & i\sin(\frac{\varphi_{\mathbf{k}}-\varphi_{\mathbf{l}}}{2}) \\ i\sin(\frac{\varphi_{\mathbf{k}}-\varphi_{\mathbf{l}}}{2}) & \cos(\frac{\varphi_{\mathbf{k}}-\varphi_{\mathbf{l}}}{2}) \end{pmatrix} \tag{B2}$$

and the explicit form of the complete interaction Hamiltonian is expressed in Eq. (A1). Because there are only intraband electron pairing involved in superconuctivity, the first four terms, the sixth term and the last term in Eq. (A1) are related to cross-band pairing and should not be involved in superconuctivity. Thus, for the Cooper pairs with the zero total momentum forming the condensate, the interaction Hamiltonian involved in superconductivity can be written as

$$\begin{aligned} H_{int} &= \frac{1}{2}\frac{1}{\Omega}\sum_{all\ \mathbf{k},\mathbf{l}}\frac{1}{2}e^{-i\zeta(\varphi_{\mathbf{k}}-\varphi_{\mathbf{l}})}V_{\mathbf{k}-\mathbf{l}}\{[1+\cos(\varphi_{\mathbf{k}}-\varphi_{\mathbf{l}})](a^{\dagger}_{\mathbf{k},+}a^{\dagger}_{-\mathbf{k},+}a_{-\mathbf{l},+}a_{\mathbf{l},+} + a^{\dagger}_{\mathbf{k},-}a^{\dagger}_{-\mathbf{k},-}a_{-\mathbf{l},-}a_{\mathbf{l},-}) \\ &\quad -[1-\cos(\varphi_{\mathbf{k}}-\varphi_{\mathbf{l}})](a^{\dagger}_{\mathbf{k},+}a^{\dagger}_{-\mathbf{k},+}a_{-\mathbf{l},-}a_{\mathbf{l},-} + a^{\dagger}_{\mathbf{k},-}a^{\dagger}_{-\mathbf{k},-}a_{-\mathbf{l},+}a_{\mathbf{l},+})\}. \end{aligned} \tag{B3}$$

This is the interaction Hamiltonian part in Eq. (14). Here we note that the signs of $\cos(\varphi_{\mathbf{p}}-\varphi_{\mathbf{p}'})$ are the same in two summations and no approximation has been made yet. Under the mean field approximation, $A_{\mathbf{l}\sigma} =< a_{-\mathbf{l}\sigma}a_{\mathbf{l}\sigma} >$, the Hamiltonian is shown in Eq. (16),

$$H_{eff} = \sum_{\mathbf{k}}\sum_{\sigma=\pm}(\epsilon_{\mathbf{k},\sigma}a^{\dagger}_{\mathbf{k},\sigma}a_{\mathbf{k},\sigma} - \frac{1}{2}\triangle_{\mathbf{k},\sigma}a^{\dagger}_{\mathbf{k},\sigma}a^{\dagger}_{-\mathbf{k},\sigma} - \frac{1}{2}\triangle^{*}_{\mathbf{k},\sigma}a_{-\mathbf{k},\sigma}a_{\mathbf{k},\sigma} + \frac{1}{2}\triangle^{*}_{\mathbf{k},\sigma}A_{\mathbf{k},\sigma}). \tag{B4}$$

where

$$\begin{aligned} \triangle_{\mathbf{k},\sigma} &= -\frac{1}{2}\frac{1}{\Omega}\sum_{\mathbf{l}}e^{-i\zeta(\varphi_{\mathbf{k}}-\varphi_{\mathbf{l}})}\{V_{\mathbf{k}-\mathbf{l}}[1+\cos(\varphi_{\mathbf{k}}-\varphi_{\mathbf{l}})]A_{\mathbf{l}\sigma} - V_{\mathbf{k}-\mathbf{l}}[1-\cos(\varphi_{\mathbf{k}}-\varphi_{\mathbf{l}})]A_{\mathbf{l}-\sigma}\} \\ &= -\frac{1}{2}\frac{1}{\Omega}\sum_{\mathbf{l}}e^{-i\zeta(\varphi_{\mathbf{k}}-\varphi_{\mathbf{l}})}\{V_{\mathbf{k}-\mathbf{l}}(A_{\mathbf{l}\sigma}-A_{\mathbf{l}-\sigma}) + V_{\mathbf{k}-\mathbf{l}}\cos(\varphi_{\mathbf{k}}-\varphi_{\mathbf{l}})(A_{\mathbf{l}\sigma}+A_{\mathbf{l}-\sigma})\}. \end{aligned} \tag{B5}$$

The effective Hamiltonian can be diagonalized by the transformation

$$\gamma_{\mathbf{k},\sigma} = u_{\mathbf{k},\sigma}a_{\mathbf{k},\sigma} - v_{\mathbf{k},\sigma}a^{\dagger}_{-\mathbf{k},\sigma} \tag{B6}$$

where $|u_{\mathbf{k},\sigma}|^2 = \frac{1}{2}(1+\frac{\epsilon_{\mathbf{k},\sigma}}{E_{\mathbf{k},\sigma}})$ and $|v_{\mathbf{k},\sigma}|^2 = \frac{1}{2}(1-\frac{\epsilon_{\mathbf{k},\sigma}}{E_{\mathbf{k},\sigma}})$. For

$$|\Psi_G>= \prod_{\substack{\mathbf{k}=\mathbf{k_1},\mathbf{k_2},\ldots,\mathbf{k_m} \\ \pi>\varphi_{\mathbf{k}_i}\geq 0}}\prod_{\sigma=+,-}(u_{\mathbf{k},\sigma} + \nu_{\mathbf{k},\sigma}a^{\dagger}_{\mathbf{k},\sigma}a^{\dagger}_{-\mathbf{k},\sigma})|\Psi_0>, \tag{B7}$$

the mean field

$$\begin{aligned} A_{\mathbf{l}\sigma} &= <\Psi_G|a_{-\mathbf{l}\sigma}a_{\mathbf{l}\sigma}|\Psi_G> \\ &= u^{*}_{\mathbf{l},\sigma}v_{\mathbf{l},\sigma} \\ &= \frac{1}{2}\frac{\triangle_{\mathbf{k},\sigma}}{E_{\mathbf{k},\sigma}}. \end{aligned} \tag{B8}$$

Thus, we obtain gap equation Eq. (24)

$$\Delta_{\mathbf{k},\sigma} = -\frac{1}{4}\frac{1}{\Omega}\sum_{all\ \mathbf{l}}V_{\mathbf{k}-\mathbf{l}}e^{-i\zeta(\varphi_{\mathbf{k}}-\varphi_{\mathbf{l}})}[(\frac{\Delta_{\mathbf{l},\sigma}}{E_{\mathbf{l},\sigma}} - \frac{\Delta_{\mathbf{l},-\sigma}}{E_{\mathbf{l},-\sigma}}) + \cos(\varphi_{\mathbf{k}}-\varphi_{\mathbf{l}})(\frac{\Delta_{\mathbf{l},\sigma}}{E_{\mathbf{l},\sigma}} + \frac{\Delta_{\mathbf{l},-\sigma}}{E_{\mathbf{l},-\sigma}})]. \tag{B9}$$

in the main text.

Appendix C: The superconducting state parameters of 2DEG from Morel and Anderson's model

In this appendix, from the analysis of the gap equation, we define dimensionless electron-phonon coupling constant λ and Coulomb psuedopotential μ^*, similiar to the analysis of Morel and Anderson [33]. These constants can be used in the presence of Rashba interaction case and expressed in terms of simple physical quantities.

1. The electron-phonon interaction and the effective interaction

The 2D model Hamiltonian for electrons with Coulomb and electron-phonon interaction is given by

$$\begin{aligned} H = &\sum_{all\ \mathbf{k}} \sum_{\sigma=\uparrow,\downarrow} \varepsilon_{\mathbf{k}} c^\dagger_{\mathbf{k}\sigma} c_{\mathbf{k}\sigma} + \sum_{all\ \mathbf{q}} \Omega_{\mathbf{q}} (b^\dagger_{\mathbf{q}} b_{-\mathbf{q}} + \frac{1}{2}) \\ &+ \frac{1}{2\Omega} \sum_{all\ \mathbf{k}',\mathbf{k},\mathbf{q}} \sum_{\sigma=\uparrow,\downarrow} V^C_{\mathbf{q}} c^\dagger_{(\mathbf{k}+\mathbf{q})\sigma} c^\dagger_{(\mathbf{k}'-\mathbf{q})\sigma'} c_{\mathbf{k}'\sigma'} c_{\mathbf{k}\sigma} \\ &+ \frac{1}{\sqrt{\Omega}} \sum_{all\ \mathbf{k}',\mathbf{k}} \sum_{\sigma=\uparrow,\downarrow} g_{(\mathbf{k}'-\mathbf{k})} [b^*_{(\mathbf{k}'-\mathbf{k})} + b_{-(\mathbf{k}'-\mathbf{k})}] c^\dagger_{\mathbf{k}'\sigma} c_{\mathbf{k}\sigma}. \end{aligned} \tag{C1}$$

The first two terms are the bare electron and bare phonon terms. $\varepsilon_{\mathbf{k}} = \frac{k^2}{2m}$ is the kinetic energy of electrons. $\Omega_{\mathbf{q}} = \sqrt{\frac{2\pi n_c Z^2 e^2}{M_c} q}$ is the 2D plasma ion frequency (Appendix C). $V^C_{\mathbf{q}} = \frac{2\pi e^2}{q+q_{TF}}$ is the 2D screened electrostatic Coulomb potential. $g_{\mathbf{q}} = -i(\frac{n_c}{2M_c\Omega_{\mathbf{q}}})^{\frac{1}{2}} qZV^C_{\mathbf{q}}$ is the electron-phonon coupling matrix element. Ω is the area of the primitive cell. $q_{TF} = 2\pi e^2 N(0)$ is the Thomas-Fermi wave vector where $N(0) = \frac{m^*}{\pi} = Zn_c/\varepsilon_F$ is the electron density of states at the the Fermi level for 2DEG. In metals, electron screening plays an important role in determining the self energy of longitudinal phonon. $\omega^2_{\mathbf{q}} = \frac{\Omega^2_{\mathbf{q}}}{\kappa}$ where κ is the 2D dielectric constant and $\omega_{\mathbf{q}}$ is the dressed longitudinal phonon frequency. Because the magnitude of phonon frequency is much smaller than the electron plasma frequency ($\sim O(\sqrt{\frac{m_e}{M_c}})$), the dielectric constant κ can be taken in the static limit, $\kappa \sim \kappa_q = 1 + \frac{q_{TF}}{q}$. The dressed longitudinal phonon frequency with Coulomb screening is

$$\omega^2_{\mathbf{q}} = \Omega^2_{\mathbf{q}}/(1 + \frac{q_{TF}}{q}) \tag{C2}$$

In the long wave limit, the dressed longitudinal phonon dispersion is

$$\begin{aligned} \omega_{\mathbf{q}} &\sim \Omega_{\mathbf{q}} (\frac{q}{q_{TF}})^{\frac{1}{2}} \\ &= (\frac{n_c Z^2}{M_c N(0)})^{\frac{1}{2}} q \\ &= cq, \end{aligned} \tag{C3}$$

This is a sound wave dispersion with sound velocity

$$c \equiv (\frac{Z}{2M_c m^*})^{\frac{1}{2}} k_F. = (\frac{m^* Z}{2M_c})^{\frac{1}{2}} v_F \tag{C4}$$

where the Fermi momentum is $k_F = (\pi n_c Z)^{\frac{1}{2}}$ and Fermi velocity is $v_F = \frac{k_F}{m^*}$. While considering Coulomb screening, the phonon frequency Ω_q in Eq. (B1) will be replaced by ω_q.

The effective interaction Hamiltonian reads

$$\begin{aligned} H_{int} = &\sum_{all\ \mathbf{q}} \omega_{\mathbf{q}} (b^\dagger_{\mathbf{q}} b_{-\mathbf{q}} + \frac{1}{2}) \\ &+ \frac{1}{\Omega} \sum_{all\ \mathbf{k}',\mathbf{k},\mathbf{q}} \sum_{\sigma=\uparrow,\downarrow} V_{\mathbf{q}} c^\dagger_{(\mathbf{k}+\mathbf{q})\sigma} c^\dagger_{(\mathbf{k}'-\mathbf{q})\sigma'} c_{\mathbf{k}'\sigma'} c_{\mathbf{k}\sigma}. \end{aligned} \tag{C5}$$

The interaction potential $V_{\mathbf{q}} = V_{\mathbf{k-l}}$ is composed of $V_{\mathbf{k,l}} = V^C_{\mathbf{k,l}} + V^{ph}_{\mathbf{k,l}}$, where the first term is the Coulomb interaction $V^C_{\mathbf{q}} = \frac{2\pi e^2}{q+q_{TF}}$ and the second term $V^{ph}_{\mathbf{k,l}}$ is the lowest order approximation of the phonon mediated electron-electron interaction

$$\begin{aligned} V^{ph}_{\mathbf{q}} &= |g_{\mathbf{q}}|^2 \frac{2\omega_q}{(\xi_{\mathbf{k}} - \xi_{\mathbf{l}})^2 - \omega_q^2} \\ &= \frac{2|g_{\mathbf{q}}|^2}{\omega_q} \frac{\omega_q^2}{(\xi_{\mathbf{k}} - \xi_{\mathbf{l}})^2 - \omega_q^2}. \end{aligned} \tag{C6}$$

2. The electron-phonon interaction contribution to the gap equation

The effective electron-electron interaction can be further simplified. We start with the electron-phonon part first

$$\begin{aligned} \frac{2|g_q|^2}{\omega_{\mathbf{q}}} &= \frac{n_c Z^2}{M_c c^2} V^{C\,2}_{\mathbf{q}} \\ &= N(0) V^{C\,2}_q \\ &= \frac{1}{N(0)} (\frac{q_{TF}}{q + q_{TF}})^2 \end{aligned} \tag{C7}$$

where we have used $N(0) = n_c Z/\varepsilon_F = \frac{n_c Z^2}{M_c(\frac{Z}{M_c}\frac{1}{2}m^* v_F^2)} = \frac{n_c Z^2}{M_c c^2}$. Here $q_{TF} = \frac{2}{a_B}\frac{m*}{m_e} \sim 3.78 m_{eff}$ (Å). It is usually much larger than Debye momentum q_D and we may replace q with an average value of Debye momentum $\frac{2}{3}q_D$ without significant error where $q_D = (4\pi n_c)^{\frac{1}{2}}$ in 2D case. From Eq. (47), for $\triangle_{\mathbf{k}} = \triangle e^{-i\zeta\varphi_{\mathbf{k}}}\cos\varphi_{\mathbf{k}}$ and approximating $\triangle_{\mathbf{k}}$ by $\triangle$ so that $E_{\mathbf{k}} \approx \sqrt{|\epsilon_{\mathbf{k}}|^2 + |\triangle|^2}$, we get the electron-phonon interaction contribution of Eq. (47) as

$$\begin{aligned} & -\frac{1}{4}\frac{1}{\Omega}\sum_{\mathbf{l}} V^{ph}_{\mathbf{kl}} \frac{\triangle_{\mathbf{l}}}{(\triangle_{\mathbf{l}}^2 + \epsilon_{\mathbf{l}}^2)^{\frac{1}{2}}} e^{-i\zeta(\varphi_{\mathbf{k}} - \varphi_{\mathbf{l}})} \cos(\varphi_{\mathbf{k}} - \varphi_{\mathbf{l}}) \\ =& -\frac{1}{4} e^{-i\zeta\varphi_{\mathbf{k}}} \int_{-\omega_D}^{\omega_D} \frac{N(0)}{2} d\epsilon_{\mathbf{l}} \int_0^{2\pi} \frac{d\varphi_{\mathbf{l}}}{2\pi} \frac{2|g_{\mathbf{q}}|^2}{\omega_q} \frac{\triangle}{(\triangle^2 + \epsilon_{\mathbf{l}}^2)^{\frac{1}{2}}} \\ & \frac{\omega_q^2}{(\epsilon_{\mathbf{k}} - \epsilon_{\mathbf{l}})^2 - \omega_q^2} \cos(\varphi_{\mathbf{k}} - \varphi_{\mathbf{l}}) \cos(\varphi_{\mathbf{l}}) \\ =& \frac{1}{8} (\frac{q_{TF}}{\frac{2}{3}q_D + q_{TF}})^2 e^{-i\zeta\varphi_{\mathbf{k}}} \int_{-\omega_D}^{\omega_D} d\epsilon_{\mathbf{l}} \frac{\triangle}{(\triangle^2 + \epsilon_l^2)^{\frac{1}{2}}} \int_0^{2\pi} \frac{d\varphi_{\mathbf{l}}}{2\pi} [1 \\ & - \frac{(\epsilon_{\mathbf{k}\sigma} - \epsilon_{\mathbf{l}\sigma'})^2}{a_{\mathbf{k}\sigma,\mathbf{l}\sigma'} + b_{\mathbf{k}\sigma,\mathbf{l}\sigma'} \cos(\varphi_{\mathbf{k}} - \varphi_{\mathbf{l}})}] \cos(\varphi_{\mathbf{k}} - \varphi_{\mathbf{l}}) \cos(\varphi_{\mathbf{l}}) \\ =& \triangle \frac{1}{2} (\frac{q_{TF}}{\frac{2}{3}q_D + q_{TF}})^2 e^{-i\zeta\varphi_{\mathbf{k}}} [\frac{1}{4} \sinh^{-1}(\frac{\hbar\omega_D}{\triangle}) \\ & - \frac{1}{4} \int_{-\omega_D}^{\omega_D} d\epsilon_{\mathbf{l}} \frac{a_{\mathbf{k}\sigma,\mathbf{l}\sigma'} (\epsilon_{\mathbf{k}\sigma} - \epsilon_{\mathbf{l}\sigma'})^2}{b^2_{\mathbf{k}\sigma,\mathbf{l}\sigma'}} \frac{\Delta_{\sigma'}}{E_{\mathbf{l},\sigma'}}] \\ =& \triangle \frac{1}{4} \lambda e^{-i\zeta\varphi_{\mathbf{k}}} [\sinh^{-1}(\frac{\hbar\omega_D}{\triangle}) \\ & - \int_{-\omega_D}^{\omega_D} d\epsilon_{\mathbf{l}} \frac{a_{\mathbf{k}\sigma,\mathbf{l}\sigma'} (\epsilon_{\mathbf{k}\sigma} - \epsilon_{\mathbf{l}\sigma'})^2}{b^2_{\mathbf{k}\sigma,\mathbf{l}\sigma'}} \frac{\Delta_{\sigma'}}{E_{\mathbf{l},\sigma'}}]. \end{aligned} \tag{C8}$$

In the above equation, we use the integration

$$\begin{aligned}
&\int_0^{2\pi}\frac{d\varphi_l}{2\pi}[\frac{(\xi_k-\xi_l)^2}{(\xi_k-\xi_l)^2-\omega_q^2}] \\
=&(\xi_k-\xi_l)^2\mathrm{P}[\int_0^{2\pi}\frac{d\varphi_l}{2\pi}\frac{1}{a+b\cos(\varphi_l-\varphi_k)}] \\
=&0 \qquad\qquad (\text{for } |a|<|b|)
\end{aligned} \tag{C9}$$

where $a=(\xi_k-\xi_l)^2-c^2(k^2+l^2)$ and $b=2c^2kl$ and k and l are close to Fermi wave number k_F, thus $|a|<|b|$. We can define dimensionless electron-phonon coupling strength parameter

$$\lambda=\frac{1}{2}(\frac{q_{TF}}{\frac{2}{3}q_D+q_{TF}})^2 \tag{C10}$$

The parameter λ plays the role of "$N(0)V$" in BCS theory as discussion of Morel and Anderson in Ref. [33] for three dimensional case. one can see that λ is a parameter that is not directly related to the coupling strength g or electron density of states for small q_D .

3. The electron-electron interaction contribution

Unlike the case of the phonon mediated processes where the energy regime is near the Fermi surface, the high energy contribution should also be considered for Coulomb interaction. Let U^C be the circular average of Coulomb potential V^C around the Fermi surface in 2D,

$$\begin{aligned}
U^C&=\int_0^{2\pi}\frac{d\varphi}{2\pi}V^C_{kl} \\
&=\int_0^{2\pi}\frac{d\varphi}{2\pi}\frac{2\pi e^2}{2k_F\sin\frac{\varphi}{2}+q_{TF}} \\
&=\frac{2\pi e^2}{q_{TF}}\frac{\frac{2}{\pi}\arccos(\frac{2k_F}{q_{TF}})}{\sqrt{1-(\frac{2k_F}{q_{TF}})^2}}.
\end{aligned} \tag{C11}$$

For small $\frac{2k_F}{q_{TF}}$,

$$\begin{aligned}
U^C&\sim\frac{2\pi e^2}{q_{TF}}\frac{\frac{2}{\pi}(\frac{\pi}{2}-\frac{2k_F}{q_{TF}})}{\sqrt{1-(\frac{2k_F}{q_{TF}})^2}} \\
&\sim\frac{2\pi e^2}{q_{TF}}(1-\frac{4}{\pi}\frac{k_F}{q_{TF}}) \\
&\sim\frac{2\pi e^2}{q_{TF}+\frac{4}{\pi}k_F}.
\end{aligned} \tag{C12}$$

Following Ref. [33],

$$\begin{aligned}
\mu&=\frac{N(0)}{2}U^C \\
&=\frac{1}{2}(\frac{q_{TF}}{q_{TF}+\frac{4}{\pi}k_F}).
\end{aligned} \tag{C13}$$

The instantaneous effective Coulomb potential $U^{C,eff}$ to be used near the Fermi surface is weaker than the Coulomb potential U^C and should be corrected as

$$U^{C,\,eff}=\frac{U^C}{1+\frac{N(0)}{2}U^C\ln(\frac{\omega_m}{\omega_D})}. \tag{C14}$$

It was first showed by Bogoliubov, Tolmachev, and Shirkov [43] and discussed subsequantly in Refs. [33,44]. Here ω_m is of the order of Fermi energy. For $\triangle_{\mathbf{k}} = \triangle e^{-i\zeta\varphi_{\mathbf{k}}} \cos\varphi_{\mathbf{k}}$ amd making the same approximation $\triangle_{\mathbf{k}} \approx \triangle$, the Coulomb interaction contribution to the gap equation Eq. (47) is

$$\begin{aligned}
&-\frac{1}{4}\frac{1}{\Omega}\sum_{\mathbf{l}} V^C_{\mathbf{kl}} e^{-i\zeta(\varphi_{\mathbf{k}}-\varphi_{\mathbf{l}})} \cos(\varphi_{\mathbf{k}}-\varphi_{\mathbf{l}}) \frac{\triangle_{\mathbf{l}}}{(\triangle_{\mathbf{l}}^2+\xi_{\mathbf{l}}^2)^{\frac{1}{2}}} \\
&= -\frac{1}{4} e^{-i\zeta\varphi_{\mathbf{k}}} \int_{-\omega_D}^{\omega_D} \frac{N(0)}{2} d\xi_{\mathbf{l}} \frac{\triangle}{(\triangle^2+\xi_{\mathbf{l}}^2)^{\frac{1}{2}}} U^{C,\,eff} \\
&\int_0^{2\pi} \frac{d\varphi}{2\pi} \cos(\varphi_{\mathbf{k}}-\varphi_{\mathbf{l}}) \cos(\varphi_{\mathbf{l}}) \\
&= -\triangle e^{-i\zeta\varphi_{\mathbf{k}}} \frac{1}{4} \mu^* \sinh^{-1}(\frac{\hbar\omega_D}{\triangle})
\end{aligned} \tag{C15}$$

where the Coulomb pseudopotential potential is

$$\begin{aligned}
\mu^* &= \frac{N(0)}{2} U^{C,\,eff} \\
&= \frac{\mu}{1+\mu\ln(\frac{\omega_m}{\omega_D})}
\end{aligned} \tag{C16}$$

as discussed in Ref. [33].

4. Energy gap and transition temperature T_C

For the isotropic s-wave in 2DEG the BCS like gap equation

$$1 = (\lambda - \mu^*) \sinh^{-1}(\frac{\hbar\omega_D}{\triangle}) \tag{C17}$$

where the phonon strength parameters λ and Coulomb strength parameter μ^* are related to Thomas-Fermi wave number q_{TF}, Debye wave number q_D, and Fermi-wave number k_F.

For the superconductivity at finite temperature T, the correspond isotropic gap equation is

$$1 = -\frac{1}{2}\sum_{all\ \mathbf{l}} V_{\mathbf{kl}} \frac{1}{(\triangle^2+\xi_l^2)^{\frac{1}{2}}} \tanh(\beta\xi_l) \tag{C18}$$

where $\beta = \frac{1}{T}$. To obtain the transition temperature T_c, we take the vanishing gap limit $\triangle \to 0$. Thus, we obtain

$$\begin{aligned}
1 &= (\lambda - \mu^*) \int_0^{\omega_D} d\xi_l \frac{1}{\xi_l} \tanh(\beta_c\xi_l) \\
&= (\lambda - \mu^*) \ln[\frac{2}{\pi}\beta_c\omega_D e^{\gamma}]
\end{aligned} \tag{C19}$$

$$T_c \simeq 1.13\omega_D \exp[-\frac{1}{(\lambda-\mu^*)}] \tag{C20}$$

For 2D free electron gas, thus $\lambda = \frac{1}{2}(\frac{q_{TF}}{q_{TF}+\frac{2}{3}q_D})^2$ and $\mu^* = \frac{\frac{1}{2}(\frac{q_{TF}}{q_{TF}+\frac{4}{\pi}k_F})}{1+\frac{1}{2}\frac{q_{TF}}{q_{TF}+\frac{4}{\pi}k_F}\ln(\frac{\varepsilon_F}{\omega_D})}$ with $q_{TF} = 3.78 m_{eff} \text{Å}^{-1}$ and$\gamma = 0.577$ is Euler's constant.

Appendix D: 2D plasma ion frequency

We discuss the 2D plasma ion frequency based on jellium model for the ions. In the jellium approximation, the nuclei charge density can be written as

$$\rho_i(\mathbf{r},t) = \rho_{0i} + \delta\rho_i(\mathbf{r},t) \tag{D1}$$

where $\rho_{0i} = ZeN_c$ is the smooth ion charge density and compensates with background electron charge resulting in charge neutral of the system. Z is the valency of the ion core and N_c is the ion area density. $\delta\rho_i(r,t)$ is the charge variation due to the ion vibration. From the continuity equation

$$\frac{\partial}{\partial t}\rho_i(\mathbf{r},t) = \nabla\cdot\mathbf{J}(\mathbf{r},t), \tag{D2}$$

we obtain

$$\begin{aligned}\frac{\partial}{\partial t}\delta\rho_i(\mathbf{r},t) &= \nabla\cdot\rho_i(\mathbf{r},t)\mathbf{v}(\mathbf{r},t)\\ &\sim \rho_{0i}\nabla\cdot\mathbf{v}(\mathbf{r},t)\end{aligned} \tag{D3}$$

While taking the time derivative of above equation, we obtain

$$\begin{aligned}\frac{\partial^2}{\partial^2 t}\delta\rho_i(\mathbf{r},t) &= \rho_{0i}\nabla\cdot\mathbf{a}(\mathbf{r},t)\\ &\sim \frac{Ze}{M_c}\rho_{i0}\nabla\cdot\mathbf{E}(\mathbf{r},t)\\ &\sim \frac{Ze}{M_c}\rho_{i0}\nabla^2\Phi(\mathbf{r},t)\end{aligned} \tag{D4}$$

where $\Phi(\mathbf{r},t)$ is the potential due to the variation charge $\delta\rho_i(\mathbf{r},t)$. The Fourier transform of $\delta\rho_i(\mathbf{r},t)$ and $\Phi(\mathbf{r},t)$ are

$$\delta\rho_i(\mathbf{r},t) = \sum_{\mathbf{q},\omega}\delta\rho_i(\mathbf{q},t)e^{-i\Omega t}e^{-i\mathbf{q}\cdot\mathbf{r}} \tag{D5}$$

and

$$\Phi(\mathbf{r},t) = \sum_{\mathbf{q},\omega}v(\mathbf{q})\delta\rho_i(\mathbf{q},t)e^{-i\Omega t}e^{-i\mathbf{q}\cdot\mathbf{r}} \tag{D6}$$

where Coulomb potential $v(\mathbf{q}) = \frac{2\pi}{q}$ for the 2D case. Substituting Eqs. (D5) and (D6) into Eq. (D4), we obtain

$$\Omega^2 = \frac{2\pi n_c Z^2 e^2}{M_c}q. \tag{D7}$$

Hence, $\Omega_q = \sqrt{\frac{2\pi n_c Z^2 e^2}{M_c}q}$ is the 2D plasma ion frequency.

Appendix E: Superconductivity of the two-band lead and free electron approximation

For bulk lead, it is reported to be a two-band superconductor. However, the strength of interband scattering is comparable to intraband scattering for the case of bulk lead so that the superconducting gaps of the two bands are very close. The superconducting gaps of bulk lead are reported to be about 1.34 meV [Supplementary ref. S1] and the difference between two superconducting gaps of bulk lead was reported to be 0.15 meV [53]. The superconductivity of lead was described very well by Eliashberg formalism [49,52]. In that and subsequent treatments, the Fermi surface was assumed to be spherical like what we have done in this work.

For the case of lead, the superconductivity would involved in the Fermi surface in second and third bands as discussed in Ref. [54]. To undestand the effect of superconductivity in the two-band lead case, we may start from the zero temperature two-band coupled gap equation [Supplementary ref. S2]

$$\Delta_{\mathbf{k},1} = -\frac{1}{2}\frac{1}{\Omega}(\sum_{all\,\mathbf{l}} V_{\mathbf{k-l},11}\frac{\Delta_{\mathbf{l},1}}{E_{\mathbf{l},1}} + \sum_{all\,\mathbf{l}} V_{\mathbf{k-l},12}\frac{\Delta_{\mathbf{l},2}}{E_{\mathbf{l},2}}) \tag{E1}$$

$$\Delta_{\mathbf{k},2} = -\frac{1}{2}\frac{1}{\Omega}(\sum_{all\,\mathbf{l}} V_{\mathbf{k-l},21}\frac{\Delta_{\mathbf{l},1}}{E_{\mathbf{l},1}} + \sum_{all\,\mathbf{l}} V_{\mathbf{k-l},22}\frac{\Delta_{\mathbf{l},2}}{E_{\mathbf{l},2}}). \tag{E2}$$

For the first gap energy $\Delta_{\mathbf{k},1}$, the first and second terms in R.H.S. of Eq. (E1) are the intraband scattering contribution and the interband scattering contribution respectively. For the second gap energy $\Delta_{\mathbf{k},1}$, the first and second terms in R.H.S. of Eq. (E2) are the interband scattering contribution and the intraband scattering contribution respectively. From the Fermi surface in Ref. [54], we assume that the two gaps are not strong momentum dependent so that $\Delta_{\mathbf{k},\delta} \sim \Delta_\delta$ and $E_{\mathbf{k},\delta} = \sqrt{\epsilon_{k,\delta}^2 + \triangle_{k,\delta}^2} \sim \sqrt{\epsilon_\delta^2 + \triangle_\delta^2} \sim E_\delta$. If the isotropic attractive interaction potential $V_{k-l,\delta\delta'} = -V_{\delta\delta'}$ is assumed, the gap equation Eqs.(E1,E2) can be written as

$$\begin{cases} \Delta_1 = & \frac{1}{2}\frac{1}{\Omega}(\sum\limits_{all\,\mathbf{l}} V_{11}\frac{\Delta_1}{E_1} + \sum\limits_{all\,\mathbf{l}} V_{12}\frac{\Delta_2}{E_2}) = \frac{1}{2}V_{11}\int_{-\hbar\omega_D}^{\hbar\omega_D} d\epsilon_1 \frac{N_1(\epsilon_1)}{E_1}\triangle_1 + \frac{1}{2}V_{12}\int_{-\hbar\omega_D}^{\hbar\omega_D} d\epsilon_2 \frac{N_2(\epsilon_2)}{E_2}\triangle_2 \\ \Delta_2 = & -\frac{1}{2}\frac{1}{\Omega}(\sum\limits_{all\,\mathbf{l}} V_{21}\frac{\Delta_1}{E_1} + \sum\limits_{all\,\mathbf{l}} V_{22}\frac{\Delta_2}{E_2}) = \frac{1}{2}V_{21}\int_{-\hbar\omega_D}^{\hbar\omega_D} d\epsilon_1 \frac{N_1(\epsilon_1)}{E_1}\triangle_1 + \frac{1}{2}V_{22}\int_{-\hbar\omega_D}^{\hbar\omega_D} d\epsilon_2 \frac{N_2(\epsilon_2)}{E_2}\triangle_2 \end{cases} \tag{E3}$$

$$\begin{cases} \Delta_1 = & N_1(0)V_{11}\triangle_1 \sinh^{-1}(\hbar\omega_D/\triangle_1) + N_2(0)V_{12}\triangle_2 \sinh^{-1}(\hbar\omega_D/\triangle_2) \\ \Delta_2 = & N_1(0)V_{21}\triangle_1 \sinh^{-1}(\hbar\omega_D/\triangle_1) + N_2(0)V_{22}\triangle_2 \sinh^{-1}(\hbar\omega_D/\triangle_2) \end{cases} \tag{E4}$$

We use the fact that the two gaps of lead are very close $\triangle_1 \sim \triangle_2 \sim \triangle$

$$\begin{cases} 1 = & N_1(0)V_{11} \sinh^{-1}(\hbar\omega_D/\triangle) + N_2(0)V_{12} \sinh^{-1}(\hbar\omega_D/\triangle) \\ 1 = & N_1(0)V_{21} \sinh^{-1}(\hbar\omega_D/\triangle) + N_2(0)V_{22} \sinh^{-1}(\hbar\omega_D/\triangle) \end{cases} \tag{E5}$$

$$\begin{cases} 1 = & [N_1(0)V_{11} + N_2(0)V_{12}] \sinh^{-1}(\hbar\omega_D/\triangle) \\ 1 = & [N_1(0)V_{21} + N_2(0)V_{22}] \sinh^{-1}(\hbar\omega_D/\triangle) \end{cases} \tag{E6}$$

$$\begin{cases} \triangle & = \frac{\hbar\omega_D}{\sinh\{1/[N_1(0)V_{11}+N_2(0)V_{12}]\}} \\ \triangle & = \frac{\hbar\omega_D}{\sinh\{1/[N_1(0)V_{21}+N_2(0)V_{22}]\}} \end{cases} \tag{E7}$$

Thus, an effective coupling strength parameter λ_{eff} may be taken as

$$\begin{aligned} \lambda_{eff} &\sim N_1(0)V_{11} + N_2(0)V_{12} \\ &\sim N_1(0)V_{21} + N_2(0)V_{22}. \end{aligned} \tag{E8}$$

If it is futher assumed that $V_{11} \sim V_{12} \sim V_{21} \sim V_{22} \sim V$,

$$\lambda_{eff} \sim [N_1(0) + N_2(0)]V \sim N_0^{eff} V \tag{E9}$$

This means that it may be approximated with an effective density of state at Fermi energy $N_0^{eff} \sim N_1(0) + N_2(0)$. For a single band case, total conduction electrons would stay in a band and form a single Fermi surface with certain density of states. For a two-band case, total conduction electrons would spread into two bands and form two separated Fermi surfaces with certain density of states. There is a connection that the total number of conduction electrons is the same and it is possible to use a single band Fermi surface to approximate two bands case. But, we know that the density of states at Fermi energy strongly depends on the bandstructure. From the Fermi surface for lead case in Ref. [54], to estimate an effective density of state at Fermi energy for second and third bands, we suggest that the free electron Fermi surface approximation would preserve the spherical symmetric property and may be a reasonable choice.

Supplementary References

S1. Giaever, I. & Megerle, K. Study of Superconductors by Electron Tunneling. *Phys. Rev.* **122**, 1101–1111 (1961).
S2. Suhl, H., Matthias, B. T. & Walker, L. R. Bardeen-Cooper-Schrieffer Theory of Superconductivity in the Case of Overlapping Bands. *Phys. Rev. Lett.* **3**, 552–554 (1959).
